# Intraocular asymmetry of visual field defects in primary angle-closure glaucoma, high-tension glaucoma, and normal-tension glaucoma in a Chinese population

**DOI:** 10.1038/s41598-021-91173-8

**Published:** 2021-06-03

**Authors:** Junhong Jiang, Cong Ye, Cong Zhang, Wenqing Ye, Xiaoyan Wang, Xiao Shang, Xiang Xu, Hongte Zhang, Shaodan Zhang, Jingwei Zheng, Jingjing Zuo, Jingjing Hu, Nathan Congdon, Fan Lu, Yuanbo Liang

**Affiliations:** 1 Eye Hospital, School of Ophthalmology and Optometry, Wenzhou Medical University, No. 270 West College Road, Wenzhou, 325027 Zhejiang China; 2 National Clinical Research Center for Ocular Disease, Wenzhou, Zhejiang China; 3 Glaucoma Institute, Wenzhou Medical University, Wenzhou, Zhejiang China; 4 Centre for Public Health, Queen’s University Belfast, Belfast, UK

**Keywords:** Eye diseases, Optic nerve diseases

## Abstract

Direct comparison data on spatial patterns of visual field (VF) defects among primary angle-closure glaucoma (PACG), high-tension glaucoma (HTG) and normal-tension glaucoma (NTG) are not available. We aimed to compare the intraocular asymmetry of VF loss among patients with PACG, NTG and HTG across different severity levels. A total of 162 eyes of 114 patients with PACG, 111 eyes of 74 patients with HTG and 148 eyes of 102 patients with NTG were included. VF defects were categorized into 3 stages (early, moderate, and advanced), and each hemifield was divided into 5 regions according to the Glaucoma hemifield test (GHT). The mean total deviation (TD) of each GHT region was calculated. In the early stage, the paracentral, peripheral arcuate 1 and peripheral arcuate 2 regions in the superior hemifield in the NTG group had significantly worse mean TDs than their corresponding regions in the inferior hemifield. In the advanced stage, the central region in the superior hemifield in the PACG group had a significantly worse mean TD than that in the inferior hemifield. There was no significant difference in the mean TD for any of the five regions between hemifields across all severity levels in the in the HTG group. The superior hemifield was affected more severely than the inferior hemifield in all three subtypes of primary glaucoma. This asymmetric tendency was more pronounced in NTG than in PACG and HTG.

## Introduction

Glaucoma is the leading global cause of irreversible blindness, affecting 79.6 million people worldwide in 2020^1^. It is a progressive optic neuropathy with characteristic structural changes and corresponding visual field (VF) defects^2^. Primary glaucoma is divided into primary angle-closure glaucoma (PACG) and primary open-angle glaucoma (POAG) based on the status of the iridocorneal angle^3^. POAG is subdivided into high-tension glaucoma (HTG) and normal-tension glaucoma (NTG).

PACG, which manifests as a crowded anterior segment and narrow anterior chamber angle, is characterized by elevated intraocular pressure (IOP) secondary to mechanical obstruction of aqueous outflow by apposition of the iris to the trabecular meshwork^4^. Pressure-dependent damage is considered to be the major pathogenesis of glaucomatous optic neuropathy in PACG patients^4^. In comparison, the mechanism of optic nerve damage in HTG is thought to be a mixture of pressure-dependent and pressure-independent causes^2^. In addition to IOP, other factors are believed to be involved in the development and worsening of HTG, such as choroidal blood flow, vascular dysregulation, and low cerebrospinal fluid pressure^5–7^. Pressure-independent vasogenic risk factors are considered to be more important in the development and progression of NTG than in HTG^6,8,9^.

The difference among PACG, HTG and NTG also reflects glaucomatous structural and functional damage. The morphometric features of glaucomatous optic nerve head damage differ between PACG, HTG and NTG. Eyes with NTG tend to have a greater degree of rim thinning, larger cup areas and cup-to-disc ratios and a smaller rim area than eyes with HTG^10,11^ and PACG^12^. Smaller optic discs with smaller cup areas and larger rim areas are more common in PACG eyes than in HTG eyes^13^.

The characteristics of VF damage in POAG have been previously reported by several studies using Goldmann perimetry; VF defects in NTG were found to be more centralized^14,15^, more localized^16–18^, steeper^14,15,19^ and more common in the superior hemifield than those in HTG^20^. In comparison, published data on VF damage in PACG are relatively limited^21^. The differences among VF defects in PACG, NTG and HTG have been reported by several studies utilizing automated perimetry; in both PACG and HTG, the superior hemifield is more severely affected than the inferior hemifield^22,23^, and the VF defects in HTG tend to be more localized than those in PACG^22,24^. However, each of these studies included only one or two types of glaucoma. Only one prior small study directly compared the interocular asymmetry of the VF defects among eyes with NTG, PACG and HTG^25^, and another study compared the VF progression rates among these 3 glaucoma subtypes^26^. Direct comparison data on the spatial patterns of VF defects among PACG, HTG and NTG are not available. We therefore compared the intraocular VF defect asymmetry among patients with PACG, NTG and HTG across different severity levels.

## Methods

### Participants

In this cross-sectional study, patients diagnosed with HTG and PACG by a glaucoma specialist (Y.B.L.) were recruited from the glaucoma clinic of the Eye Hospital of Wenzhou Medical University from January 2017 to December 2019. Patients with NTG were recruited from the Wenzhou Glaucoma Progression Study (WGPS), a longitudinal community-based study providing free glaucoma screenings in the Wenzhou area^27,28^. Written informed consent was obtained from all participants. The current study was approved by the Ethics Committee of the Eye Hospital of Wenzhou Medical University, and adhered to the tenets of the Declaration of Helsinki.

All participants in the current study had PACG, NTG or HTG. Glaucoma was defined according to the criteria of the International Society for Geographical and Epidemiological Ophthalmology (ISGEO) and the Collaborative Normal-Tension Glaucoma Study^3,8^. PACG was defined as the presence of angle closure together with evidence of glaucomatous optic neuropathy and a corresponding VF defect, and angle closure was defined as the inability to visualize the posterior trabecular meshwork for ≥ 180° on gonioscopy. Primary open-angle glaucoma (POAG) defined as the presence of an open anterior chamber angle as assessed by gonioscopy, and evidence of glaucomatous optic neuropathy and a corresponding VF defect. POAG patients with six untreated IOP measurements consistently < 21 mmHg, with no single measurement > 24 mmHg and no more than one reading equal to 23 or 24 mmHg were classified as normal-tension glaucoma (NTG), and others as high-tension glaucoma (HTG)^8^. Glaucomatous optic neuropathy was defined as the presence of any of the following: optic disc hemorrhage, retinal nerve fiber layer (RNFL) defect, vertical cup-to-disc ratio (CDR) > 0.7 and/or CDR asymmetry > 0.2, or a neuroretinal rim width < 0.1^3^. Glaucomatous VF defects were defined according to Hodapp‑Parrish‑Anderson’s criteria^29^.

Additional inclusion criteria were as follows: age ≥ 18 years, presenting visual acuity ≥ 6/18, and a spherical equivalent (SE) refractive error between − 6.0 and + 3.0 diopter (D). Patients were excluded if they had secondary glaucoma, previous laser or incisional surgery of the retina, and/or other conditions potentially affecting the visual field.

Each potential participant underwent a comprehensive ophthalmic examination by a certified ophthalmic technician, including assessment of presenting visual acuity, refraction, IOP, slit-lamp biomicroscopy, gonioscopy, fundus photography (Visucam 200; Carl Zeiss Meditec, Inc., Dublin, CA, USA), and standard automated perimetry (Humphrey Field Analyzer [HFA] II; Carl Zeiss Meditec, Inc.). IOP was measured between 8:00 AM and 5:00 PM on one day and the median of two readings was used.

VF examinations were performed with the white-on-white 24–2 Swedish interactive threshold algorithm (SITA) program. VF tests with fixation loss rates < 20% or false-positive and false negative error rates < 15% were considered reliable and eligible for analysis; only reliable VF tests having glaucoma hemifield test (GHT) outside normal limits were included and the first VF test for each participant was excluded from the analysis. VF severity was categorized into three stages based on the mean deviation (MD): early glaucoma (≥ −6 dB), moderate glaucoma (< − 6 dB and > − 12 dB), and advanced glaucoma (≤ − 12 dB)^30^. To evaluate the pattern of VF defects, the total deviation (TD) probability plot was divided into five subfield regions in each of the superior and inferior hemifields: central, paracentral, nasal, and two peripheral (arcuate 1 and arcuate 2), derived from the GHT^23,31,32^. These GHT sectors were divided based on normal retinal nerve fiber layer anatomy^33^. When recording pointwise data and dividing regions, VF tests of left eyes were inverted to resemble those of right eyes for ease of comparison. The mean TD values of the 10 visual field regions were calculated, including both superior and inferior hemifields.

### Statistical analysis

Because 45.2% of the participants had both eyes included in current study, generalized estimating equation (GEE) models^34^ were used to adjust for correlations between both eyes of a subject in the analysis of data related to eyes. The GEE model was used to compare the demographic characteristics of the groups. For the pointwise analysis, the mean TD value of each VF test point in the superior hemifield was compared with its corresponding point in the inferior hemifield at each severity level for the three glaucoma groups using the GEE model, accounting for mean TD. For the regional analysis, the mean TDs of the 5 GHT regions in the superior hemifield were compared with their counterparts in the inferior hemifield at each severity level in the three groups using the GEE model, with adjustments for mean TD and sex. The relationship between MD and pattern standard deviation (PSD) was compared in the three groups using GEE model. Bonferoni correction was used to adjust for multiple comparisons of VF defects. Triplets of matched patients from each of the 3 glaucoma subtypes (PACG, NTG and HTG) were generated using propensity-score matching. The propensity score was calculated using multivariable logistic regression analysis based on the severity of VF defects, age, sex and MD. Statistical significance was set at P < 0.05, and all analyses were performed using SPSS software version 21.0 (IBM., Chicago, IL) and “R” software (R version 4.0.2).

## Results

### Comparisons among glaucoma subtypes

One hundred and sixty-two eyes of 114 participants with PACG, 111 eyes of 74 participants with HTG and 148 eyes of 102 participants with NTG were enrolled in this study (Table 1). Participants with HTG were significantly younger than those with NTG and PACG (HTG vs. NTG, P = 0.012; HTG vs. PACG, P < 0.001). There were more women than men in the PACG and NTG groups, while there were more men in the HTG group. The mean SE refraction in the PACG group was significantly more hyperopic (positive) than that in the NTG and HTG groups (both P < 0.001). The mean IOP in the NTG group was significantly lower than that in the HTG group (P < 0.001). LogMAR VA in the PACG group was significantly better than that in the NTG group (P < 0.001).

**Table 1:** Demographic characteristics of participants.

Subtypes	Characteristics	Total	Early	Moderate	Advanced	P
PACG	Eyes, n	162	57	31	74	
	Age (year)	62.6 ± 9.3	60.9 ± 10.5	64.3 ± 8.1	63.3 ± 8.7	0.501
	Male Gender, n (%)	51(44.7%)	18(41.9%)	9(39.1%)	24(50.0%)	0.275
	VA (logMAR)	0.16 ± 0.16	0.12 ± 0.14	0.11 ± 0.14	0.21 ± 0.16	**0.001**
	SE (D)	0.16 ± 1.49	−0.01 ± 1.90	0.58 ± 1.11	0.11 ± 1.24	0.127
	IOP (mmHg)	16.63 ± 7.90	14.68 ± 6.31	16.37 ± 5.52	18.25 ± 9.43	**0.039**
	MD (dB)	−13.88 ± 10.77	−3.10 ± 1.81	−8.51 ± 1.69	−24.44 ± 6.00	** < 0.001**
	PSD (dB)	6.13 ± 3.50	3.12 ± 1.54	7.74 ± 2.74	7.76 ± 3.35	** < 0.001**
	VFI (%)	61.57 ± 35.13	95.14 ± 3.07	80.87 ± 7.07	27.62 ± 22.09	** < 0.001**
HTG	Eyes, n	111	24	16	71	
	Age (year)	54.7 ± 15.6	47.6 ± 18.0	63.2 ± 15.1	55.4 ± 13.4	**0.002**
	Male gender, n (%)	50(67.6%)	14(73.7%)	7(58.3%)	29(67.4%)	0.102
	VA (logMAR)	0.20 ± 0.15	0.14 ± 0.16	0.19 ± 0.17	0.22 ± 0.14	0.126
	SE (D)	−1.36 ± 2.36	−1.63 ± 2.78	−1.36 ± 2.90	−1.27 ± 2.10	0.840
	IOP (mmHg)	18.70 ± 8.14	18.34 ± 5.07	16.68 ± 4.62	19.27 ± 9.48	0.195
	MD (dB)	−16.79 ± 10.17	−2.62 ± 2.33	−9.12 ± 1.93	−23.31 ± 5.88	** < 0.001**
	PSD (dB)	8.01 ± 3.99	3.94 ± 2.51	8.93 ± 4.47	9.18 ± 3.38	** < 0.001**
	VFI (%)	50.68 ± 32.78	94.08 ± 5.59	77.63 ± 9.23	29.94 ± 20.30	** < 0.001**
NTG	Eyes, n	148	92	36	20	
	Age (year)	62.8 ± 13.1	63.1 ± 11.9	65.4 ± 11.7	56.8 ± 19.2	0.185
	Male gender, n (%)	49(48.0%)	34(51.5%)	8(34.8%)	7(53.9%)	0.935
	VA (logMAR)	0.20 ± 0.15	0.19 ± 0.14	0.19 ± 0.16	0.25 ± 0.15	0.255
	SE (D)	−1.36 ± 3.00	−1.05 ± 2.81	−1.36 ± 3.25	−2.76 ± 3.15	0.157
	IOP (mmHg)	14.38 ± 3.50	14.66 ± 3.56	14.18 ± 3.27	13.44 ± 3.59	0.443
	MD (dB)	−6.17 ± 5.28	−3.00 ± 1.94	−8.42 ± 1.58	−16.72 ± 4.21	** < 0.001**
	PSD (dB)	6.60 ± 3.98	4.41 ± 2.39	8.98 ± 3.30	12.40 ± 2.39	** < 0.001**
	VFI (%)	83.75 ± 15.61	92.68 ± 4.92	77.97 ± 6.86	53.05 ± 15.53	** < 0.001**

Data are mean ± standard deviation unless otherwise indicated.

*VA* visual acuity, *SE* spherical equivalent, *IOP* intraocular pressure, *TD* total deviation, *MD* mean deviation, *PSD* pattern standard deviation, *VFI* visual field index.

Bold values: P < 0.05

In the early and moderate stages, there were no significant differences in the MD and mean TD among the PACG, NTG and HTG groups. In the advanced stage, the MD and mean TD in the HTG and PACG groups were significantly worse than those in the NTG group (all P < 0.05). There were no significant differences in age or sex across the early, moderate, and advanced severity levels among the PACG, HTG and NTG groups (Table 1). In the PACG group, VA was worst and IOP was highest in the advanced stage (all P < 0.05); SE was similar across all severity levels. In the HTG and NTG groups, VA, IOP and SE were similar across all severity levels.

Of the 421 eyes included in this study, 144 eyes were propensity-matched into triplets, with each glaucoma subtype comprising 48 eyes. There was no significant difference in age, sex, MD or degree of VF loss among the PACG, HTG and NTG groups (P = 0.154, 0.310, 0.272, 0.644, respectively, Table 2).

**Table 2:** Demographic characteristics of the matched participants.

Variable	PACG	HTG	NTG	P
Eyes, n	48	48	48	
Age (year)	61.2 (7.98)	56.1 (17.3)	58.8 (15.9)	0.154
Male sex, n (%)	16 (38.1%)	25 (61.0%)	20 (46.5%)	0.310
MD (dB)	−7.40 (5.99)	−9.76 (8.74)	^-^8.04 (7.26)	0.272
**VF defects severity, n (%)**				0.644
Early stage	24(50.0%)	21(43.8%)	24(50.0%)	
Moderate stage	15(31.2%)	14(29.2%)	12(25.0%)	
Advanced stage	9(18.8%)	13(27.1%)	12(25.0%)	

Data are mean ± standard deviation unless otherwise indicated.

*MD* mean deviation, *VF* visual field.

### Comparisons between hemifields

**Figure 1:**
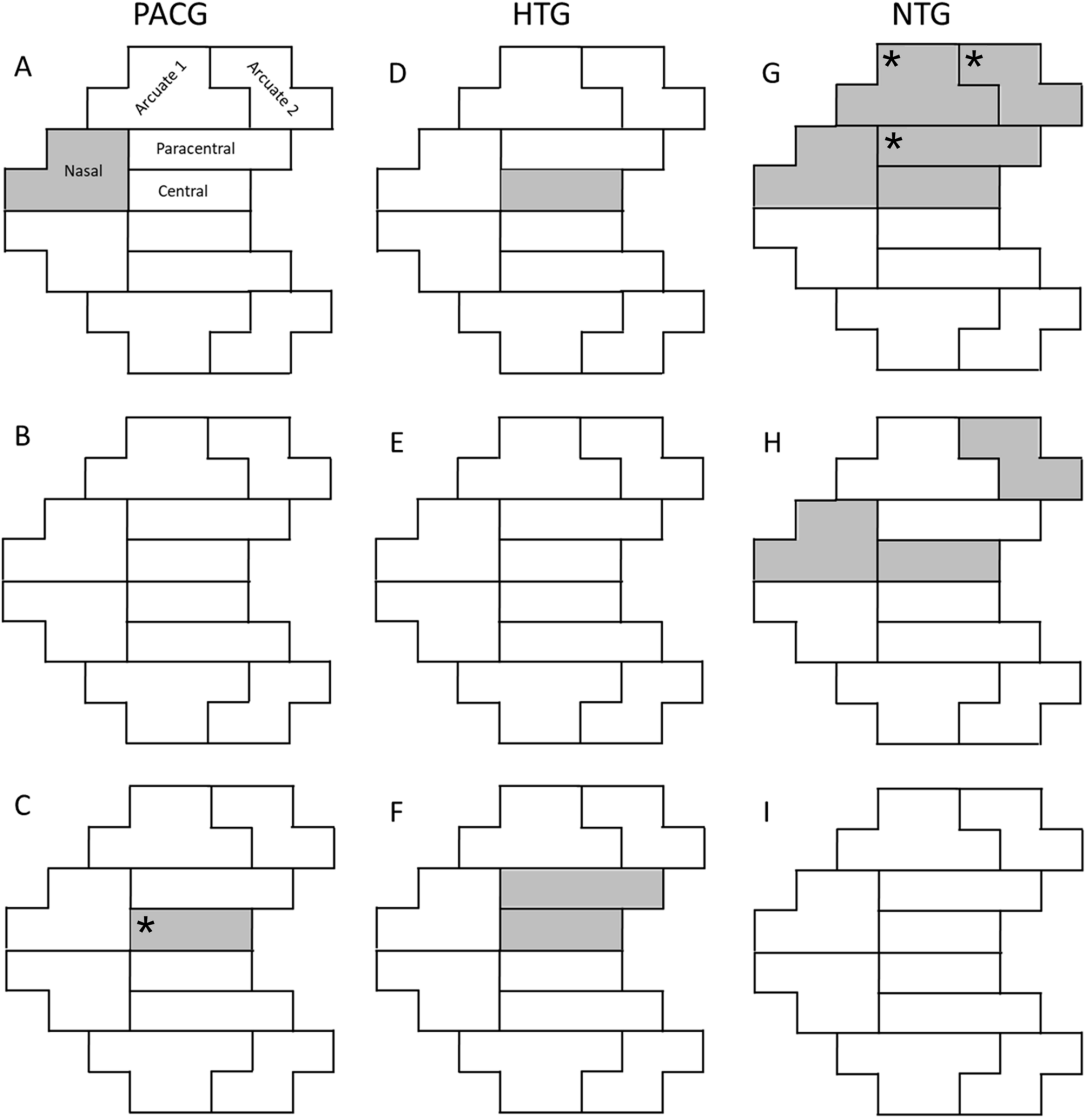
Between-hemifield comparisons of the GHT regions across severity levels. **(A–C)** show the superior-inferior asymmetry of VF defects in the PACG group. **(D–F)** show the HTG group. **(G–I)** show the NTG group. **(A,D,G)** represent early stages; **(B,E,H)** represent moderate stages, and **(C,F,I)** represent advanced stages. The shaded regions indicate significantly worse TD values than their inferior counterparts (P < 0.05) and asterisks indicate significant regions after Bonferoni correction. Image was generated using Microsoft PowerPoint software (Version 2016, Microsoft, Redmond, WA).

In the early stage, the mean TD of the superior nasal region in the PACG group was significantly worse than that of the inferior hemifield (P = 0.032, Table 3, Fig. 1A). However, there was no significant difference between the hemifields in the remaining four regions. In the early-stage HTG group (Fig. 1D), the central region in the superior hemifield had a significantly worse mean TD than that in the inferior hemifield (P = 0.022); the remaining four regions were not significantly different between hemifields. In the early-stage NTG group, all five GHT regions in the superior hemifield had significantly worse mean TDs than those in the inferior hemifield (Fig. 1G). In the moderate stage, three superior hemifield regions (nasal, central, and peripheral arcuate 2) in the NTG group also had significantly worse mean TDs than their corresponding regions in the inferior hemifield (all P < 0.05, Fig. 1H). There were no significant differences in the mean TDs of the five regions between hemifields in the moderate-stage of the HTG and PACG groups (all P > 0.05, Fig. 1B,E). In the advanced-stage PACG group, the superior hemifield central region had a significantly worse mean TD than that in the inferior hemifield (P < 0.001, Fig. 1C); in the advanced-stage HTG group, both the central and paracentral regions of the superior hemifield had significantly worse mean TDs than those in the inferior hemifield (P = 0.015 and P = 0.045, respectively, Fig. 1F). There was no significant difference in the mean TD for any of the five regions between hemifields in the advanced-stage NTG group. The mean TDs were significantly worse in the superior hemifield in the early- and moderate-stage NTG groups (all P < 0.05), while there were no significant differences between hemifields across all severity levels in the PACG and HTG groups. After Bonferoni correction, in the early stage, the paracentral, peripheral arcuate 1 and peripheral arcuate 2 regions in the superior hemifield in the NTG group had significantly worse mean TDs than their corresponding regions in the inferior hemifield (all P < 0.05, Table 3). In the advanced stage, the central region in the superior hemifield in the PACG group had a significantly worse mean TD than that in the inferior hemifield. (P = 0.00021, after Bonferoni correction).

**Table 3:** Glaucoma hemifield test region pattern deviation for participants.

PACG, n = 162		Early, n = 57		Moderate, n = 31		Advanced, n = 74	
Region	Subregion	Mean TD	P (P*)	Mean TD	P (P*)	Mean TD	P (P*)
Central	Superior	−2.84 ± 3.13	0.850(1.00)	−7.63 ± 7.33	0.376(1.00)	−23.28 ± 9.45	< 0.001(0.0002)
	Inferior	−2.92 ± 3.77		−6.02 ± 6.60		−19.10 ± 9.47	
Paracentral	Superior	−2.97 ± 2.31	0.276(1.00)	−10.44 ± 8.39	0.145(1.00)	−25.83 ± 8.12	0.187(1.00)
	Inferior	−2.63 ± 2.16		−7.13 ± 4.37		−24.43 ± 8.98	
Nasal	Superior	−4.60 ± 4.30	0.032(0.478)	−14.03 ± 7.53	0.177(1.00)	−27.12 ± 6.11	0.579(1.00)
	Inferior	−3.49 ± 2.56		−11.04 ± 6.82		−26.71 ± 6.94	
Peripheral, arcuate 1	Superior	−3.72 ± 3.32	0.394(1.00)	−10.23 ± 5.62	0.087(1.00)	−25.95 ± 5.62	0.856(1.00)
	Inferior	−3.39 ± 2.80		−7.76 ± 4.49		−25.79 ± 8.00	
Peripheral, arcuate 2	Superior	−3.65 ± 4.15	0.339(1.00)	−9.38 ± 6.90	0.259(1.00)	−24.75 ± 6.38	0.640(1.00)
	Inferior	−3.15 ± 2.61		−7.69 ± 5.33		−25.23 ± 7.91	
Hemifield	Superior	−3.65 ± 2.72	0.305(1.00)	−10.62 ± 4.67	0.150(1.00)	−25.61 ± 6.18	0.247(1.00)
	Inferior	−3.17 ± 2.12		−8.14 ± 3.80		−24.74 ± 6.91	

HTG, n = 111		Early, n = 24		Moderate, n = 16		Advanced, n = 71	
Region	Subregion	Mean TD	P (P*)	Mean TD	P (P*)	Mean TD	P (P*)
Central	Superior	−3.51 ± 5.83	0.022(0.328)	−10.19 ± 9.77	0.767(1.00)	−23.83 ± 8.55	0.015(0.23)
	Inferior	−1.35 ± 2.12		−8.98 ± 9.60		−20.22 ± 10.56	
Paracentral	Superior	−3.21 ± 3.99	0.394(1.00)	−8.66 ± 8.46	0.775(1.00)	−27.12 ± 7.69	0.045(0.671)
	Inferior	−2.63 ± 2.93		−9.73 ± 8.80		−24.53 ± 9.46	
Nasal	Superior	−4.29 ± 6.33	0.050(0.751)	−13.56 ± 9.16	0.664(1.00)	−25.20 ± 6.87	0.253(1.00)
	Inferior	−1.88 ± 3.13		−12.00 ± 7.43		−23.88 ± 8.53	
Peripheral, arcuate 1	Superior	−2.54 ± 3.34	0.792(1.00)	−7.80 ± 6.57	0.643(1.00)	−24.12 ± 7.98	0.647(1.00)
	Inferior	−2.36 ± 3.13		−9.19 ± 7.41		−23.48 ± 9.17	
Peripheral, arcuate 2	Superior	−3.08 ± 5.13	0.814(1.00)	−6.91 ± 7.11	0.604(1.00)	−23.21 ± 7.90	0.397(1.00)
	Inferior	−2.82 ± 3.56		−8.09 ± 6.54		−22.00 ± 10.23	
Hemifield	Superior	−3.29 ± 3.98	0.214(1.00)	−9.43 ± 6.46	0.915(1.00)	−24.71 ± 6.23	0.112(1.00)
	Inferior	−2.24 ± 2.57		−9.70 ± 6.38		−23.05 ± 8.33	

NTG, n = 148		Early, n = 92		Moderate, n = 36		Advanced, n = 20	
Region	Subregion	Mean TD	P (P*)	Mean TD	P (P*)	Mean TD	P (P*)
Central	Superior	−2.76 ± 4.02	0.039(0.581)	−10.87 ± 9.32	0.008(0.119)	−14.38 ± 10.43	0.646(1.00)
	Inferior	−1.83 ± 2.95		−5.22 ± 4.36		−16.03 ± 11.56	
Paracentral	Superior	−4.15 ± 4.89	< 0.001(0.002)	−10.70 ± 8.92	0.165(1.00)	−16.80 ± 12.55	0.666(1.00)
	Inferior	−2.27 ± 3.27		−7.34 ± 7.55		−18.73 ± 11.37	
Nasal	Superior	−5.32 ± 5.46	0.045(0.673)	−12.87 ± 9.20	0.028(0.417)	−20.18 ± 10.71	0.647(1.00)
	Inferior	−3.77 ± 5.46		−7.84 ± 7.95		−21.83 ± 9.63	
Peripheral, arcuate 1	Superior	−4.89 ± 4.49	< 0.001(< 0.001)	−10.07 ± 6.54	0.077(1.00)	−17.33 ± 12.39	0.823(1.00)
	Inferior	−2.12 ± 2.57		−6.71 ± 6.37		−18.28 ± 9.30	
Peripheral, arcuate 2	Superior	−4.74 ± 5.72	< 0.001(< 0.001)	−9.84 ± 7.36	< 0.001(0.595)	−14.68 ± 11.98	0.452(1.00)
	Inferior	−1.13 ± 1.99		−6.31 ± 7.22		−13.41 ± 10.03	
Hemifield	Superior	−4.54 ± 3.63	< 0.001(< 0.001)	−10.89 ± 5.85	0.028(0.358)	−17.00 ± 10.47	0.756(1.00)
	Inferior	−2.30 ± 2.60		−6.80 ± 5.34		−17.98 ± 7.29	

*TD* total deviation.

* P values after Bonferoni correction. Data are mean ± standard deviation unless otherwise indicated.

**Figure 2:**
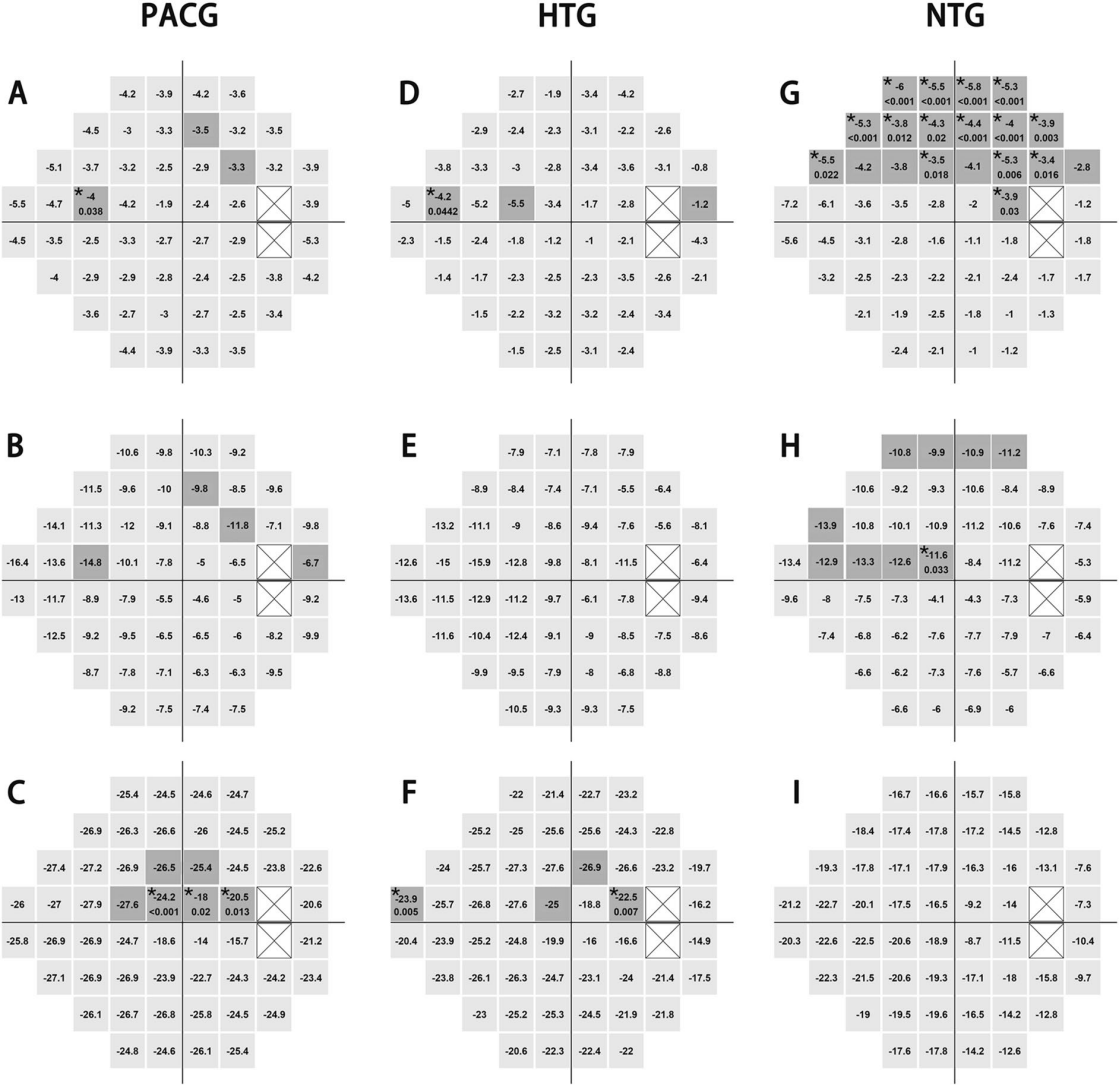
Between-hemifield comparisons of pointwise locations across severity levels. **(A–C)** show the patterns of visual field defects in the PACG group. **(D–F)** show the HTG group, **(G–I)** show the NTG group; **(A,D,G)** represent early stages; **(B,E,H)** represent moderate stages, and **(C,F,I)** represent advanced stages. The dark-shadow points indicate significantly worse TD values than their corresponding locations in the inferior hemifield (P < 0.05) and asterisks indicate significant points after Bonferoni correction. Image was generated using “R” software (R version 4.0.2, http://www.r-project.org).

Figure 2 shows the pointwise comparisons between the superior and inferior hemifields among PACG, HTG and NTG groups. In the early-stage PACG group, one in the nasal region of the superior hemifield had a significantly worse TD than its corresponding point in the inferior hemifield (P = 0.038, after Bonferoni correction, Fig. 2A). In the early-stage HTG group, one point in the nasal region had a significantly worse TD than its corresponding point in the inferior hemifield (P = 0.0442, after Bonferoni correction, Fig. 2D). In the early-stage NTG group, one point in the nasal region, two points in the paracentral region and several points clustered in the arcuate 1 and arcuate 2 regions had significantly worse TDs than their corresponding points in the inferior hemifield after Bonferoni correction (all P < 0.05, Fig. 2G). In the moderate-stage PACG group, one point each in the nasal, paracentral and peripheral arcuate 1 regions, and one point in the region adjacent to the blind spot in the superior hemifield had significantly worse mean TDs than their counterparts in the inferior hemifield, while these significant differences were disappeared after Bonferoni correction (all P > 0.05, Fig. 2B). In the moderate-stage NTG group, one point in the central region in the superior hemifield had a significantly worse TD than its corresponding point in the inferior hemifield (P = 0.033, after Bonferoni correction, Fig. 2H). In the advanced-stage PACG group, two points in the central region and one point in the region adjacent to the blind spot in the superior hemifield had significantly worse TDs than their corresponding points in the inferior hemifield after Bonferoni correction (all P < 0.05, Fig. 2C). In the advanced-stage HTG group, one point in the nasal region and one point in the region adjacent to the blind spot in the superior hemifield had significantly worse TDs than their corresponding points in the inferior hemifield (P = 0.005 and 0.007, respectively, after Bonferoni correction, Fig. 2F). There was no significant difference in the mean TD for any points between the hemifields in either the moderate-stage HTG group (Fig. 2E) or advanced-stage NTG group (Fig. 2I).

**Figure 3:**
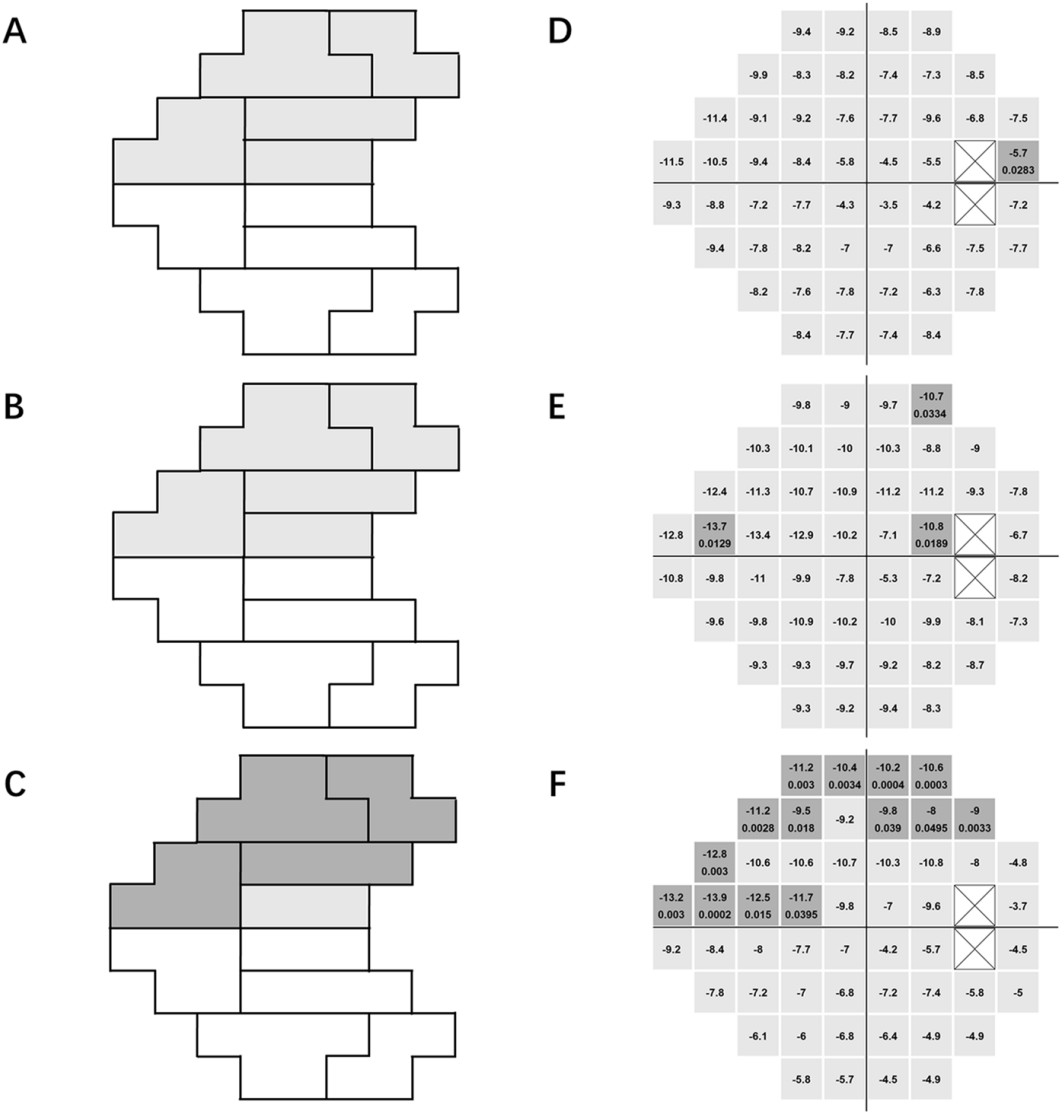
Between-hemifield comparisons of GHT regions and pointwise locations among the matched subjects. **(A–C)** show the comparisons of regions. **(D–F)** show the comparisons of pointwise locations. **(A,D)** represent PACG; **(B,E)** represent HTG, and **(C,F)** represent NTG. The dark-shadow regions and dark-shadow points represent significantly worse TD values than their inferior hemifield counterparts (P < 0.05). Image was generated using Microsoft PowerPoint software (Version 2016, Microsoft, Redmond, WA) and “R” software (R version 4.0.2, http://www.r-project.org).

Figure 3 shows the comparisons between the superior and inferior hemifields for the matched subjects. The mean TD of the superior hemifield, as a whole, was worse than that of the inferior hemifield, and this difference was most significant in the NTG group (P = 0.243, 0.250 and 0.002 for PACG, HTG and NTG, respectively). In the PACG and HTG groups, all 5 GHT regions in the superior hemifield had worse mean TDs than that that of their counterparts in the inferior hemifield; however, the differences were not statistically significant (all P > 0.05, Fig. 3A,B). In the NTG group, the paracentral, nasal, arcuate 1 and arcuate 2 regions in the superior hemifield had significantly worse mean TDs than their counterparts in the inferior hemifield (P = 0.045, 0.003, 0.007 and 0.001, respectively, Fig. 3C).

In the PACG group, one point in the region adjacent to the blind spot had a significantly worse TD than its corresponding point in the inferior hemifield (Fig. 3D). In the HTG group, one point in the nasal region, one point in the arcuate 2 region and one point in the region adjacent to the blind spot in the superior hemifield had significantly worse TDs than their corresponding points in the inferior hemifield (Fig. 3E). In the NTG group, one point in the central region and several points clustered in the nasal, arcuate 1 and arcuate 2 regions had significantly worse TDs than their corresponding points in the inferior hemifield (Fig. 3F).

### Relationship between PSD and MD

**Figure 4:**
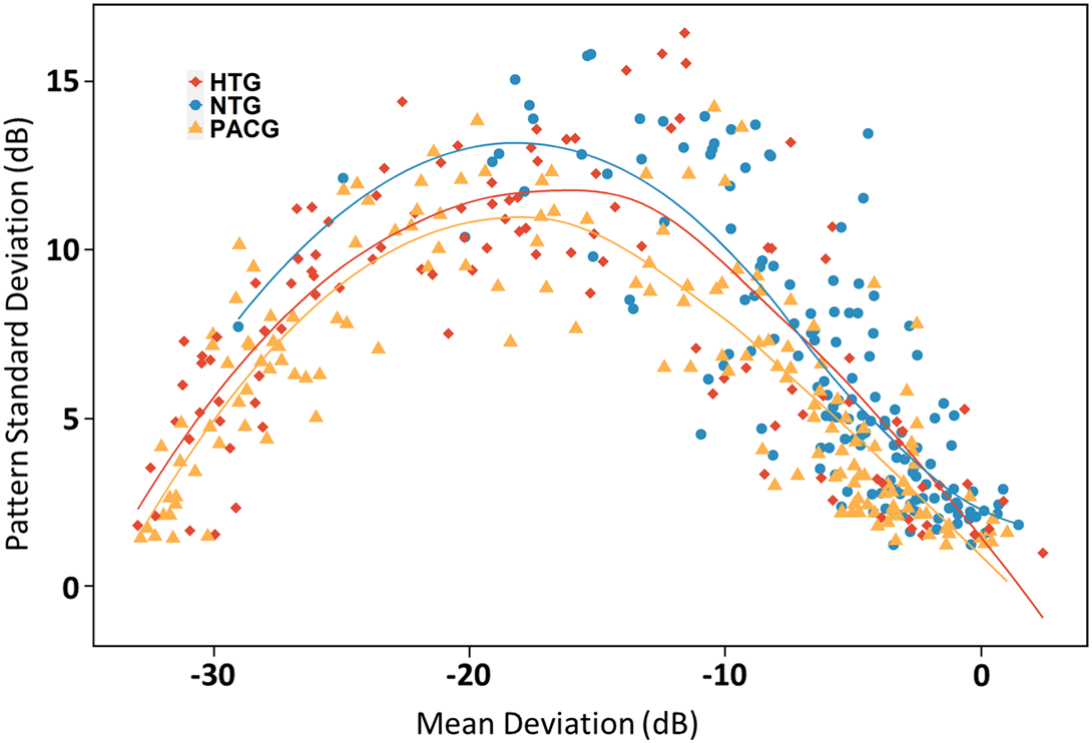
Scatterplot of PSD versus MD for eyes with PACG, HTG, and NTG. Lines represent best-fit quadratic functions for each group. Yellow triangles represent PACG eyes; red squares represent HTG eyes; blue circles represent NTG eyes. Image was generated using “R” software (R version 4.0.2, http://www.r-project.org).

The relationships between PSD and MD in the three groups followed an inverted-U shape, demonstrating that PSD worsens as MD worsens until the damage is so extensive that the PSD begins to decline (Fig. 4). The best-fit quadratic curves for the NTG, HTG, and PACG groups demonstrated that the NTG group had higher PSD values and the PACG group had lower PSD values for a given MD. After controlling for MD and (MD)^2^, the PACG group had significantly lower PSD values for a given MD than either the NTG or HTG group (PACG vs. NTG, P = 0.016; PACG vs. HTG, P < 0.001, after Bonferoni correction).

## Discussion

In early-stage PACG eyes in the current study, the nasal region in the superior hemifield had significantly worse VF damage than that in the inferior hemifield region. These results are consistent with reports by Bonomi et al^35^ based on 53 eyes with acute angle-closure glaucoma attacks and Lau et al^36^ on early-stage PACG eyes. On the other hand, Gazzard et al^23^ found that the central region in advanced-stage PACG eyes had significantly greater damage in the superior hemifield than in the inferior hemifield. Atalay et al^31^ reported that five regions in the superior hemifield had significantly worse MDs than their counterparts in the inferior hemifield in advanced-stage PACG eyes. Yousefi et al^32^ also observed more severe damage in the central and peripheral (arcuate 2) regions of advanced-stage PACG eyes. These three studies are in agreement with our finding that the central region in advanced-stage PACG eyes had significantly worse VF damage in the superior hemifield than in the inferior hemifield.

In HTG eyes in the current study, the central region in the early stage and the central and paracentral regions in the advanced stage had significantly greater damage in the superior hemifield than in the inferior hemifield. This result is similar to that in the report by Gazzard and associates^23^, who compared the characteristics of VF defects between HTG and PACG eyes. Their early-stage HTG eyes had significantly lower sensitivity in the paracentral region of the superior hemifield than in the inferior hemifield; advanced-stage HTG eyes had significantly lower sensitivity in the superior central region. In early-stage and advanced-stage HTG eyes in both Gazzard et al^23^ and the current study, only the central and paracentral regions in the superior hemifield were more damaged than the corresponding regions in the inferior hemifield. However, in a previous study conducted by Yousefi et al^32^, almost all superior GHT regions had significantly worse VF damage than the corresponding inferior regions in Japanese POAG patients. The main reason for the discordant intraocular asymmetric VF defect patterns among these studies is likely the different criteria used in defining POAG. In Yousefi’s^32^ study, POAG was defined as the presence of glaucomatous optic neuropathy with an open anterior chamber angle, while IOP was not a diagnostic criterion. The proportion of NTG among POAG cases in the Japanese population is as high as 92%;^37^ thus, a number of NTG cases may have been included in the POAG group in the Yousefi study, which may have influenced VF defect patterns in POAG eyes.

In NTG eyes, five regions in the early stage and three regions in the moderate stage had significantly greater damage in the superior hemifield than in the inferior hemifield. Park et al^38^ evaluated the patterns of VF defects in 34 NTG eyes by dividing probability plots into 2 subfields for each of the hemifields. They found that the depth of VF defects in the superior paracentral area was greater than that in the corresponding inferior area, which is consistent with the results of our study. As described above, Yousefi et al^32^ assessed VF damage among Japanese patients with POAG, among whom many likely had NTG. They observed that three GHT regions in the early stage, and five GHT regions in the moderate and advanced stages had significantly worse VF damage than the corresponding regions in the inferior hemifield. This is in agreement with our findings, to a certain extent. Araie et al^20^ compared the differences in VF characteristics between 68 early NTG and 62 early HTG. In their study, VF defects in NTG were more common in the superior hemifield than those in HTG, and in their early-stage NTG group, several points clustered in the nasal, central, paracentral, arcuate 1 and arcuate 2 regions in the superior hemifield had worse TDs than their corresponding points in the inferior hemifield^20^, which is consist with our study. In the NTG group of this study, VF defects were more common in the superior hemifield than the inferior hemifield; however, this asymmetric tendency disappeared with worsening disease severity. This might be due to the change of vascular function or the structural alteration in optic disc during progression of glaucoma, which is difficult to verify in this cross-sectional study. Further prospective studies are needed.

In the current study, PACG patients were more likely to be female than HTG patients. This is consistent with previous studies suggesting that women are at greater risk of PACG than men^39–41^. The PACG patients were significantly older than the HTG patients, also consistent with previous population-based studies suggesting that older age is a strong risk factor for PACG^40,42–44^. Patients with NTG in this study were recruited from a community screening program for subjects aged 50 years or older^27^. This may partly explain why the NTG patients were significantly older than the HTG patients. A higher rate of hyperopic SE refraction was observed in the PACG group than in the NTG and HTG groups, which is in agreement with previous reports describing the strong association between hyperopia and PACG, whereas myopia is reported to be associated with POAG^32,45,46^. Patients with NTG recruited from a community screening program had significantly better VF parameters and were more likely to be in the early or moderate stage of glaucoma than patients with HTG or NTG. This finding is in accordance with prior reports, including our own involving this screening cohort, which found that glaucoma patients detected by screening had significantly milder VF damage than those initially diagnosed in the clinic^27,47^.

The MDs of regions in the superior hemifield were worse than those of regions in the inferior hemifield in the three primary glaucoma subtypes groups; this result is in accordance with those of previous studies. Caprioli et al^15^ evaluated the VF of patients with NTG and HTG by computerized perimetry (Octopus programs, 30-degree visual field) and found that the densest scotomas occurred more frequently in the superior hemifield in both groups. Heijl et al^48^ evaluated the distribution of VF loss in HTG patients using automated perimetry and found superior VF loss to be more common than inferior VF loss. McNaught et al^49^ also reported similar intraocular VF damage asymmetry in patients with PACG. This tendency towards vertically asymmetric VF defects has also been demonstrated in studies using static automated perimetry^23,31,32^. Retinal ganglion cell axons converge at the optic nerve head, travel through the lamina cribrosa, and enter the optic nerve^50^. The structural changes in the optic nerve head and lamina cribrosa result in a corresponding functional loss of VF, and eyes with lamina cribrosa defects in the inferior half of the optic nerve head have worse VF loss in the superior hemifield^51^. Intraocular asymmetry of VF defects is likely related to the pattern of susceptibility of the optic nerve head. Previous studies have demonstrated that the inferior temporal optic nerve head has a lower collagen density than other regions^52^, rendering it more susceptible to damage during the onset and progression of glaucoma. Consistent with in vitro studies, the inferotemporal region of the optic nerve head has greater susceptibility to glaucomatous damage than other areas. Caprioli et al^11^ found that patients with POAG had greater thinning of the neuroretinal rim in the inferior and inferotemporal regions. Nouri-Mahdavi et al^22^ also reported a higher prevalence of localized rim loss in the inferotemporal sector of the optic disc in patients with POAG and PACG. Such structural differences likely underlie the greater vulnerability of the superior VF to glaucomatous damage.

The different patterns of superior-inferior asymmetry in VF defects in the three glaucoma subtypes groups may be associated with different pathogenic mechanisms. PACG is principally an IOP-dependent glaucoma that develops due to elevated IOP secondary to angle closure^4^. The mechanism of HTG is thought to be mixed, but VF damage is most closely linked to IOP^53^. IOP-independent mechanisms including vasogenic risk factors are likely to play a more significant role in the pathogenesis of glaucomatous optic neuropathy in NTG than in HTG^6,8,9^. Mechanisms of VF damage caused by IOP-dependent factors may be different from those caused by IOP-independent factors, and this may explain the observed differences in the patterns of VF defects among NTG, PACG and HTG patients in the current and other studies. Furthermore, studies on the morphologic characteristics of the optic nerve head have found differences between the 3 subtypes of glaucoma: NTG eyes had a larger cup and smaller rim than HTG^10,11,54,55^ and PACG eyes^12^. These different patterns of glaucomatous optic neuropathy may be a further indication of different pathogenic mechanisms of glaucoma damage in patients with PACG, HTG and NTG.

In the current study, PACG eyes had a lower PSD than NTG and HTG eyes. This finding agrees with previous studies reporting that VF loss was more diffuse in PACG eyes than in POAG eyes at the same level of overall field damage^22–24,32^. The NTG eyes in the current study had a higher PSD than the HTG eyes for a given MD, which is consistent with previous reports that POAG with a lower IOP tends to have more localized field defects than eyes with a higher IOP^16^.

The strengths of our study include the fact that it is one of the first to compare the patterns of VF defects between patients with PACG and POAG in China. Over the last two decades, data on this important topic from China have been limited, with only a few small studies^23,25,31^, only one of which included patients with NTG; however, it focused on only interocular asymmetry and included few patients (42 NTG, 38 POAG, and 37 CACG)^25^. The NTG patients in the current report were recruited from a longitudinal, community-based study, which may strengthen the generalizability of the findings.

Limitations of this study must also be acknowledged. First, the HTG and PACG patients were recruited from clinical settings, which may have led to the inclusion of HTG and PACG patients who were more severely affected than the NTG patients identified in the community. Second, although we excluded patients with vision impairment or blindness, prevalent cataracts may have affected the pattern of observed VF defects. Finally, the study was cross-sectional in design; a longitudinal design is needed to determine the actual pattern of progression in the different subtypes of primary glaucoma.

In summary, we found that the superior hemifield was affected more severely than the inferior hemifield in all three subtypes of primary glaucoma, and this tendency was more pronounced in NTG compared to PACG and HTG. Moreover, the VF damage in NTG and HTG was more localized than that in PACG.

## References

[1] Quigley HA, Broman AT (2006). The number of people with glaucoma worldwide in 2010 and 2020. Br. J. Ophthalmol..

[2] Weinreb RN, Khaw PT (2004). Primary open-angle glaucoma. Lancet.

[3] Foster PJ, Buhrmann R, Quigley HA, Johnson GJ (2002). The definition and classification of glaucoma in prevalence surveys. Br. J. Ophthalmol..

[4] Sun X (2017). Primary angle closure glaucoma: What we know and what we don't know. Prog. Retin. Eye. Res..

[5] Drance SM (1972). Some factors in the production of low tension glaucoma. Br. J. Ophthalmol..

[6] Flammer J (2002). The impact of ocular blood flow in glaucoma. Prog. Retin. Eye. Res..

[7] Wang N (2012). Orbital cerebrospinal fluid space in glaucoma: the Beijing intracranial and intraocular pressure (iCOP) study. Ophthalmology.

[8] Comparison of glaucomatous progression between untreated patients with normal-tension glaucoma and patients with therapeutically reduced intraocular pressures. Collaborative Normal-Tension Glaucoma Study Group. *Am. J. Ophthalmol.* **126**, 487–497. 10.1016/s0002-9394(98)00223-2 (1998). 10.1016/s0002-9394(98)00223-2 9780093

[9] Flammer J, Mozaffarieh M (2007). What is the present pathogenetic concept of glaucomatous optic neuropathy?. Surv. Ophthalmol..

[10] Eid TE, Spaeth GL, Moster MR, Augsburger JJ (1997). Quantitative differences between the optic nerve head and peripapillary retina in low-tension and high-tension primary open-angle glaucoma. Am. J. Ophthalmol..

[11] Caprioli J, Spaeth GL (1985). Comparison of the optic nerve head in high- and low-tension glaucoma. Arch. Ophthalmol..

[12] Zhao L, Wu L, Wang X (2009). Optic nerve head morphologic characteristics in chronic angle-closure glaucoma and normal-tension glaucoma. J. Glaucoma..

[13] Sihota R, Sony P, Gupta V, Dada T, Singh R (2005). Comparing glaucomatous optic neuropathy in primary open angle and chronic primary angle closure glaucoma eyes by optical coherence tomography. Ophthalmic. Physiol. Opt..

[14] Hitchings RA, Anderton SA (1983). A comparative study of visual field defects seen in patients with low-tension glaucoma and chronic simple glaucoma. Br. J. Ophthalmol..

[15] Caprioli J, Spaeth GL (1984). Comparison of visual field defects in the low-tension glaucomas with those in the high-tension glaucomas. Am. J. Ophthalmol..

[16] Caprioli J, Sears M, Miller JM (1987). Patterns of early visual field loss in open-angle glaucoma. Am. J. Ophthalmol..

[17] Chauhan BC, Drance SM, Douglas GR, Johnson CA (1989). Visual field damage in normal-tension and high-tension glaucoma. Am. J. Ophthalmol..

[18] Drance SM, Douglas GR, Airaksinen PJ, Schulzer M, Hitchings RA (1987). Diffuse visual field loss in chronic open-angle and low-tension glaucoma. Am. J. Ophthalmol..

[19] Levene RZ (1980). Low tension glaucoma: a critical review and new material. Surv. Ophthalmol..

[20] Araie M, Yamagami J, Suziki Y (1993). Visual field defects in normal-tension and high-tension glaucoma. Ophthalmology.

[21] Douglas, G. R., Drance, S. M. & Schulzer, M. The visual field and nerve head in angle-closure glaucoma. A comparison of the effects of acute and chronic angle closure. *Arch. Ophthalmol.* **93**, 409–411. 10.1001/archopht.1975.01010020423004 (1975). 10.1001/archopht.1975.01010020423004 1131080

[22] Rhee K, Kim YY, Nam DH, Jung HR (2001). Comparison of visual field defects between primary open-angle glaucoma and chronic primary angle-closure glaucoma in the early or moderate stage of the disease. Korean. J. Ophthalmol..

[23] Gazzard G (2002). The severity and spatial distribution of visual field defects in primary glaucoma: a comparison of primary open-angle glaucoma and primary angle-closure glaucoma. Arch. Ophthalmol..

[24] Boland MV, Zhang L, Broman AT, Jampel HD, Quigley HA (2008). Comparison of optic nerve head topography and visual field in eyes with open-angle and angle-closure glaucoma. Ophthalmology.

[25] Huang P, Shi Y, Wang X, Liu M, Zhang C (2014). Interocular asymmetry of the visual field defects in newly diagnosed normal-tension glaucoma, primary open-angle glaucoma, and chronic angle-closure glaucoma. J. Glaucoma..

[26] Ballae Ganeshrao S, Senthil S, Choudhari N, Sri Durgam S, Garudadri CS (2019). Comparison of visual field progression rates among the high tension glaucoma, primary angle closure glaucoma, and normal tension glaucoma. Invest. Ophthalmol. Vis. Sci..

[27] Liang Y (2018). Effect of community screening on the demographic makeup and clinical severity of glaucoma patients receiving care in urban China. Am. J. Ophthalmol..

[28] Lin S (2019). Parapapillary choroidal microvasculature dropout is associated with the decrease in retinal nerve fiber layer thickness: A prospective study. Invest. Ophthalmol. Vis. Sci..

[29] Hodapp, E., Parrish, R. K. II. & Anderson, D. R. *Clinical Decisions in Glaucoma*. (C.V. Mosby Co, 1993).

[30] Brusini P, Johnson CA (2007). Staging functional damage in glaucoma: review of different classification methods. Surv. Ophthalmol..

[31] Atalay E (2016). Pattern of visual field loss in primary angle-closure glaucoma across different severity levels. Ophthalmology.

[32] Yousefi S (2018). Asymmetric patterns of visual field defect in primary open-angle and primary angle-closure glaucoma. Invest. Ophthalmol. Vis. Sci..

[33] Asman P, Heijl A (1992). Glaucoma hemifield test. Automated visual field evaluation. Arch Ophthalmol..

[34] Huang J, Huang J, Chen Y, Ying GS (2018). Evaluation of approaches to analyzing continuous correlated eye data when sample size is small. Ophthalmic. Epidemiol..

[35] Bonomi L, Marraffa M, Marchini G, Canali N (1999). Perimetric defects after a single acute angle-closure glaucoma attack. Graefes. Arch. Clin. Exp. Ophthalmol..

[36] Lau LI, Liu CJ, Chou JC, Hsu WM, Liu JH (2003). Patterns of visual field defects in chronic angle-closure glaucoma with different disease severity. Ophthalmology.

[37] Iwase A (2004). The prevalence of primary open-angle glaucoma in Japanese: The Tajimi Study. Ophthalmology.

[38] Park JH, Yoo C, Park J, Kim YY (2017). Visual field defects in young patients with open-angle glaucoma: Comparison between high-tension and normal-tension glaucoma. J. Glaucoma..

[39] Foster, P. J. *et al.* Glaucoma in Mongolia. A population-based survey in Hövsgöl province, northern Mongolia. *Arch. Ophthalmol.* **114**, 1235–1241. 10.1001/archopht.1996.01100140435011 (1994). 10.1001/archopht.1996.01100140435011 8859083

[40] Liang Y (2011). Prevalence and characteristics of primary angle-closure diseases in a rural adult Chinese population: The Handan Eye Study. Invest. Ophthalmol. Vis. Sci..

[41] He M (2006). Prevalence and clinical characteristics of glaucoma in adult Chinese: A population-based study in Liwan District, Guangzhou. Invest. Ophthalmol. Vis. Sci..

[42] Liang YB (2011). Prevalence of primary open angle glaucoma in a rural adult Chinese population: The Handan eye study. Invest. Ophthalmol. Vis. Sci..

[43] Qu W (2011). Prevalence and risk factors for angle-closure disease in a rural Northeast China population: a population-based survey in Bin County, Harbin. Acta. Ophthalmol..

[44] Sun J (2012). Prevalence and risk factors for primary open-angle glaucoma in a rural northeast China population: A population-based survey in Bin County, Harbin. Eye.

[45] Shen L (2016). The association of refractive error with glaucoma in a multiethnic population. Ophthalmology.

[46] Gordon, M. O. *et al.* The ocular hypertension treatment study: Baseline factors that predict the onset of primary open-angle glaucoma. *Arch. Ophthalmol.*** 120**, 714–720; discussion 829–730. 10.1001/archopht.120.6.714 (2002). 10.1001/archopht.120.6.714 12049575

[47] Grodum K, Heijl A, Bengtsson B (2002). A comparison of glaucoma patients identified through mass screening and in routine clinical practice. Acta. Ophthalmol. Scand..

[48] Heijl, A. & Lundqvist, L. The frequency distribution of earliest glaucomatous visual field defects documented by automatic perimetry. *Acta Ophthalmol. (Copenh).* **62**, 658–664. 10.1111/j.1755-3768.1984.tb03979.x (1984). 10.1111/j.1755-3768.1984.tb03979.x 6485761

[49] McNaught EI, Rennie A, McClure E, Chisholm IA (1974). Pattern of visual damage after acute angle-closure glaucoma. Trans. Ophthalmol. Soc. U. K..

[50] Weinreb RN, Aung T, Medeiros FA (2014). The pathophysiology and treatment of glaucoma: A review. JAMA.

[51] Kiumehr S (2012). In vivo evaluation of focal lamina cribrosa defects in glaucoma. Arch. Ophthalmol..

[52] Winkler M (2010). High resolution three-dimensional reconstruction of the collagenous matrix of the human optic nerve head. Brain Res. Bull..

[53] Chauhan BC, Drance SM (1990). The influence of intraocular pressure on visual field damage in patients with normal-tension and high-tension glaucoma. Invest. Ophthalmol. Vis. Sci..

[54] Yamagami J, Araie M, Shirato S (1992). A comparative study of optic nerve head in low- and high-tension glaucomas. Graefes. Arch. Clin. Exp. Ophthalmol..

[55] Kiriyama N (2003). A comparison of optic disc topographic parameters in patients with primary open angle glaucoma, normal tension glaucoma, and ocular hypertension. Graefes. Arch. Clin. Exp. Ophthalmol..

